# The place of dexmedetomidine light sedation in patients with acute brain injury

**DOI:** 10.1186/s13054-019-2637-9

**Published:** 2019-11-01

**Authors:** Simone Carelli, Gennaro De Pascale, Nicoletta Filetici, Maria Grazia Bocci, Gian Marco Maresca, Salvatore Lucio Cutuli, Cecilia Maria Pizzo, Giuseppe Bello, Luca Montini, Anselmo Caricato, Giorgio Conti, Massimo Antonelli

**Affiliations:** 1 Istituto di Anestesiologia e Rianimazione, Università Cattolica del Sacro Cuore, Rome, Italy; 2 Dipartimento di Scienze dell’Emergenza, Anestesiologiche e della Rianimazione, Fondazione Policlinico Universitario A. Gemelli IRCCS, Rome, Italy

To the Editor,

An individualized titration of sedative and analgesic drugs is pivotal in the late phase management of acute brain injury (ABI) patients, when weaning from mechanical ventilation (MV) needs to be implemented [1]. Due to its pharmacologic profile, dexmedetomidine (Dex) represents a drug of choice in such setting. Nevertheless, its use in ABI patients has been recently debated mainly as a consequence of its hemodynamic effects [2, 3]. The present study aimed to evaluate clinical outcomes and safety profile of Dex administration in this patients’ category.

We retrospectively analysed prospectively collected data on the main clinical features and adverse events observed during light sedation with dexmedetomidine (Dex-LS) in ICU patients with ABI. Light sedation was defined by the maintenance of a Richmond Agitation and Sedation Scale (RASS) score between 1 and − 2. The rate of potential side effects during Dex-LS was compared with the 6-h period before Dex initiation (see Additional file 1 for further details).

The main clinical and analgosedation characteristics of the 101 included patients are listed in Table 1. Traumatic ABI (77.2%) was the main admission diagnosis, and haemorrhage (59.4% of the cohort) was the most common admission feature (see Additional file 1: Table S1). Out of 101 patients, 80 were mechanically ventilated during Dex-LS. In most cases, dexmedetomidine was administered in association with other sedatives, opioids or antipsycotic drugs, for a median duration and dosage of 64 h and 0.6 μg/kg/h, respectively.

**Table 1:** Features of 101 patients undergoing Dex-LS

Clinical characteristics			
Age, years	53 [35–68]		
Male	84 (83.2)		
Neuro-psychiatric comorbidities	24 (23.8)		
- Preesistent psychosis	12 (11.9)		
- Preesistent dementia	5 (5)		
- Preesistent epilepsy	7 (6.9)		
Traumatic brain injury (TBI)	78 (77.2)		
- Isolated traumatic brain injury	14 (13.8)		
- Polytrauma with traumatic brain injury	64 (63.4)		
Non-traumatic brain injury	23 (22.8)		
ISS at admission (TBI only)	23 [17–29]		
Head-AIS at admission (TBI only)	3 [2–4]		
GCS at admission*	10 [7–14]		
SAPS II at Dex-LS start	34 [26–44]		
SOFA at Dex-LS start	4 [3–7]		
MV at admission	91 (90.1)		
MV at Dex-LS start	80 (79.2)		
ICU LOS pre-Dex-LS, days	4 [2–8]		
ICU LOS post-Dex-LS, days	8 [3–15]		
ICU mortality	4 (4)		
Hospital mortality	8 (7.9)		
Hours of MV^#^	39 [12–72]		
MV in assisted mode^#^	71 (88.8)		
MV in assisted mode, hours^#^	24 [6–48]		
Successful weaning^#^	56 (70)		
Successful weaning in pts with previous weaning failure^##^	16 (57.1)		
Spontaneous breathing, hours^#^	25 [0–72]		
Analgosedation details			
RASS	0 [− 1/0]		
Propofol co-infusion	35 (34.7)		
Midazolam co-infusion	0		
Remifentanil co-infusion	59 (58.4)		
Other opioids co-infusion**	15 (14.9)		
Antipsychotic drugs co-administration***	40 (39.6)		
Dex length of infusion, hours	64 [33–120]		
Dex start dosage, μg/kg/h	0.7 [0.5–0.9]		
Dex median dosage, μg/kg/h	0.6 [0.5–0.9]		
Dex maximum dosage, μg/kg/h	0.9 [0.6–1.2]		
Dex dosage at suspension, μg/kg/h	0.5 [0.3–0.8]		
Hemodynamic parameters and adverse events (*n* = 101)			
	Pre-Dex infusion^###^	Dex-LS	*P* value
RASS	− 2 [− 3/0]	0 [− 1/0]	< 0.001
HR, bpm	78 [70–89]	80 [66–91]	0.165
SAP, mmHg	133 [124–146]	139 [126–150]	0.136
DAP, mmHg	65 [56–72]	69 [62–78]	< 0.001
MAP, mmHg	85 [78–98]	90 [84–99]	0.039
Bradycardia	2 (2)	23 (22.8)	< 0.001
- Bradycardia in pts receiving remifentanil co-infusion^^^	–	12 (20.3)	–
- Dex median dosage in pts with bradycardia, μg/kg/h^^^^	–	0.6 [0.4–0.9]	–
Arterial hypotension requiring vasopressors	42 (41.6)	27 (26.7)	0.037
Seizures	3 (3)	3 (3)	1
ICP, mmHg*	9 [8–14]	8 [7–10]	0.164

*Dex* dexmedetomidine, *LS* light sedation, *ISS* Injury Severity Score, *AIS* Abbreviated Injury Scale, *GCS* Glasgow Coma Scale, *SAPS II* Simplified Acute Physiology Score II, *SOFA* Sequential Organ Failure Assessment, *MV* mechanical ventilation, *LOS* length of stay, *RASS* Richmond Agitation-Sedation Scale, *HR* heart rate, *SAP* systolic arterial pressure, *DAP* diastolic arterial pressure, *MAP* mean arterial pressure, *ICP* intracranial pressure, *pts* patients

Data are shown as median [IQR] or *N* (%)

*GCS at admission was available in 96 patients. ICP was monitored in 10 patients

**Sufentanil, morphine

***Haloperidol, quetiapine, chlorpromazine

^#^Eighty out of 101 patients were mechanically ventilated during Dex-LS

^##^Twenty-eight patients failed at least a weaning attempt before Dex-LS

^###^Data of 6-h pre-Dex infusion period were analysed

^^^Fifty-nine patients received remifentanil co-infusion

^^^^Twenty-three patients had bradycardia event(s)

Dexmedetomidine has been administered safely in our population of ABI patients. Dex infusion rate and duration were comparable with those previously described [2, 3]. The rate of systemic arterial hypotension was consistent with available findings [2, 3] and lower compared with the pre-infusion period. The 23% rate of bradycardia takes place in the wide range of occurrence reported in ABI patients [2]. Nevertheless, bradycardia never imposed dexmedetomidine interruption. These findings should be interpreted in the light of the relatively young age and low severity scores of our population, where Dex was frequently co-infused with other sedatives or opioids. Neither seizure rate nor intracranial pressure increased during Dex-LS, supporting the clinical absence of Dex impact on cerebral physiology [4].

During Dex-LS, the majority of patients were weaned from MV, including more than half who previously failed a weaning attempt. These observations are in line with the available evidence comparing Dex sedation with midazolam and propofol use, even though in ICU patients without ABI [5].

In conclusion, despite the intrinsic limitations of our retrospective design lacking a control group, this study suggests that when used to target light sedation in our cohort of ABI patients, dexmedetomidine was safe and enabled the weaning from MV and the maintenance of spontaneous breathing.

## Supplementary information

**Additional file 1.** Electronic Supplementary Material.

## Data Availability

The datasets used and/or analysed during the current study are available from the corresponding author on reasonable request.
