# Supplementary material for: The place of dexmedetomidine light sedation in patients with acute brain injury
Source: Crit Care. 2019 Nov 1;23:340. doi: 10.1186/s13054-019-2637-9 (PMC6825350; doi:10.1186/s13054-019-2637-9)
Supplement: Supplementary file 1 — Additional file 1. Electronic Supplementary Material. [file 13054_2019_2637_MOESM1_ESM.docx]

**The place of dexmedetomidine light sedation in patients with acute brain injury**

**Electronic Supplementary Material**

*Study design and inclusion criteria*.

We performed a retrospective analysis on data prospectively collected from electronic ICU charts (Digistat®) on the use of dexmedetomidine light sedation in patients with acute brain injury admitted to two Intensive Care Units (ICUs) (18-bed general ICU and 10-bed neuro-ICU) of a tertiary teaching hospital in Italy, that admit approximately 1000 patients/year. The study was approved by the local ethics committee (no. UCSC34998/18) and because of its observational, non-interventional design, informed consent was waived. Eligibility criteria were as follows: admission between February 2014 and September 2018 with a diagnosis of acute brain injury, either traumatic or not; no need of deep sedation and controlled mechanical ventilation as part of a neuroprotection strategy; use of dexmedetomidine for at least 12 consecutive hours; length of ICU stay ≥ 72 hours; full availability of clinical data.

Referring to hemodynamic, neurological variables and adverse events, data acquired during dexmedetomdine infusion were compared to a 6-hours pre-infusion period, the latter including propofol as main sedative agent and remifentanil/sufentanil for analgesia. Dexmedetomidine was infused according to recommended dosages, without starting bolus or dose tapering over 0.5 mcg/kg/h. Sedation depth was evaluated according to the Richmond Agitation Sedation Scale (RASS) [1].

*Adverse events.* Bradycardia was defined as a heart rate < 50 bpm. We recorded systemic arterial hypotension events when requiring vasopressors infusion. Seizures were defined by the occurrence of any clinical and/or instrumental evidence of epileptic activity. In patients receiving intracranial pressure continuous monitoring, median values before and during dexmedetomidine infusion were recorded (see Table 1 for further details).

*Statistic analysis*. The Kolmogorov–Smirnov test was used to evaluate the distribution of variables. Categorical variables were given as proportions. Data of pre-infusion and dexmedetomidine light sedation periods were compared with the Fisher’s exact test or Wilcoxon Test, as appropriate. *p* < 0.05 was considered significant. All statistical analyses were performed using MedCalc software, version 12.2.1 (MedCalc®, MariaKerke, Belgium).

**Table S1** Brain injury features and mechanisms

| **Brain injury type** | **N (%)** |
| --- | --- |
| Subarachnoid haemorrhage | 39 (38.6) |
| Subdural hematoma | 29 (28.7) |
| Intraparenchymal haemorrhage | 26 (25.7) |
| Contusions | 13 (12.9) |
| Epidural hematoma | 10 (9.9) |
| Epilepsy | 8 (7.9) |
| Penetrating injury | 7 (6.9) |
| Ischemic stroke | 7 (6.9) |
| Intraventricular haemorrhage | 6 (5.9) |
| Encephalitis | 3 (3) |
| Meningitis | 3 (3) |
| Pneumocephalus | 2 (2) |

***References***

1. Sessler CN, Gosnell MS, Grap MJ, Brophy GM, O’Neal P V., Keane KA, et al. The Richmond Agitation-Sedation Scale: Validity and reliability in adult intensive care unit patients. Am J Respir Crit Care Med. 2002;166:1338–44.
